# Health financing and systems in African small and island states: Unique challenges and opportunities in achieving universal health coverage

**DOI:** 10.1016/j.ssmhs.2025.100104

**Published:** 2025-12

**Authors:** Finn McGuire, Sakshi Mohan, Megha Rao, Juliet Nabyonga-Orem, Ajoy Nundoochan, Issiaka Sombie, Edward Kataika, Simon Bland, Paul Revill

**Affiliations:** a Centre for Health Economics, University of York, United Kingdom; b World Health Organisation, Namibia Country Office, Namibia; c Centre for Health Professions Education, Faculty of Health Sciences, North-West University, South Africa; d World Health Organisation, Mauritius Country Office, Mauritius; e West African Health Organisation, Burkina Faso; f East, Central and Southern African Health Community, Tanzania; g Global Institute for Disease Elimination, United Arab Emirates

**Keywords:** Comparative health systems, Health financing, UHC, Small and island states

## Abstract

While Africa has made substantial health progress, small and island states face distinct vulnerabilities and threats, demanding focused attention. Employing WHO building blocks, this study explores the health systems and financing status of small and island states in Africa, emphasizing their unique challenges in achieving universal health coverage. We undertake a comparative analysis of health systems and financing between African small and island states and larger counterparts within the region. Despite their unique challenges, African small and island states appear to perform comparatively well both in terms of health financing and for a number of key health system inputs. These findings suggest that the hypothesized structural impediments facing small and island states may be less severe than anticipated, or that good policies may have effectively mitigated these challenges within the health sector. However, many small and island states remain understudied, and further health research must be undertaken to better understand the nuances of health systems in these countries.

## Highlights

• This paper outlines the challenges in financing and operating sustainable health systems in small and island states (S&IS).
• First comparative analysis of the health system performance of African S&IS.
• Despite challenges, African S&IS have higher total health expenditure per capita and government prioritisation of health spending.
• Findings suggest structural impediments affecting S&IS health sector's may be less severe than anticipated, or offset by good policies.
• However, S&IS remain understudied and further health research must be undertaken.

## 1 Introduction

Continued progress towards achieving universal health coverage (UHC) will require increased prioritisation of health by governments and accompanying sustained growth in public health expenditure. Estimates suggest, to attain the health targets of the Sustainable Development Goals (SDGs), low- and middle-income countries (LMICs) must increase spending on health as a proportion of gross domestic product (GDP) from a current mean of 5.6–7.5 % (Stenberg et al., 2017). Further, successive global economic, health and political shocks have amplified questions about the sustainability and resilience of LMICs health systems, while emerging environmental threats and the changing global health financing landscape threaten to undermine progress made. Small and island states (S&IS) health systems may be particularly exposed and vulnerable to this increasing instability and changing global circumstances.

Country ‘size’ has long been recognised as an important characteristic, presenting advantages and disadvantages (Kuznets, 1960, Benedict, 1967, Selwyn, 1980, Srinivasan, 1986, Streeten, 1993, Alesina and Spolaore, 1997). In particular, Economic Geography brought attention to issues associated with size and location, primarily focusing on economic growth, trade and development (Easterly and Kraay, 2000, Collier and Dollar, 1999, Aiyar, 2008, Brito, 2015; World Development Report, 2009). As a group, S&IS are frequently cited as having idiosyncratic challenges which may impede economic development, as well as being particularly susceptible to exogenous shocks (Briguglio, 1995, Briguglio, 2014, Baldacchino and Bertram, 2009).^1^ These issues, have led to what is described as the ‘small-country problem’ (Collier, 2007). Specifically, S&IS are characterised by their “narrow economic base, high production costs, shortage of skilled labour and heavy dependence on trade and foreign aid” (Suzana et al., 2018), all of which have a direct or indirect impact on the health sector. This recognition of the distinctive characteristics and challenges of S&IS has resulted in the formation of special interest groups and multilateral initiatives (Appendix A).

Despite growing interest, relatively limited attention has been paid to the health and health systems of S&IS. Much of the literature has centred on specific health conditions of which S&IS face disproportionately high burdens of e.g. non-communicable diseases (NCDs) specifically weight-related conditions such as diabetes and cardiovascular disease (Samuels, 2019) or increasingly the link between climate and health in S&IS (Tukuitonga and Vivili, 2021). Existing research on health systems in small states has often taken a Euro-centric focus (WHO, 2014). This dearth of evidence on health systems in LMIC S&IS is particularly stark given the importance of human capital to S&IS. While social development is important for all countries, it is especially so for S&IS which must often compensate for limited natural resource endowments with high quality human capital (Briguglio, 2022, World Health Organisation, 2023). Given the importance of health in human capital formation (Becker, 1964, Grossman, 1972), investments in health have a significant impact on economic performance (Bloom and Canning, 2003, Bloom et al., 2019). Together, this suggests that the potential economic benefits from investing in health may be larger in smaller countries.

This study examines the health financing status, health systems and unique challenges and opportunity which affect progress towards UHC of African S&IS. The study has two key objectives. First, we undertake a narrative review of existing literature examining aspects of health systems for which African S&IS face unique challenges and justify distinct examination of this country grouping from a health perspective. Second, using a quantitative descriptive analysis, we provide an overview of the current state of health financing, health systems and progress towards UHC in African S&IS. This is done by placing African S&IS within the wider regional context. This is particularly important given the continental health policy commitments outlined in the African Union Development Agency New Partnership for Africa’s Development (AUDA-NEPAD) African Health Strategy (AHS) 2016–2030. The AHS aims to “strengthen health systems performance, increase investments in health, improve equity and address social determinants of health to reduce priority disease burdens by 2030” (African Union, 2016). While S&IS may be overlooked at the expense of their larger counterparts from a research perspective, the AHS alongside the 2019 African Union – African Leadership Meeting (AU-ALM) commitments to health financing to accelerate progress towards UHC, does not prioritise between countries with each theoretically holding equal status. The ALM highlighted four pillars for health financing; secure ‘more money for health’, achieve ‘more health for the money’, ensure ‘equity’ and financial risk protection, and strengthen ‘leadership and governance’. These commitments require supporting all AU member countries, regardless of size.

Section 2 provides a brief definition of S&IS and outlines the countries of focus. Section 3 provides a brief overview of the methods and data used. Section 4 provides a short review of the literature on S&IS describing the general idiosyncratic characteristics, challenges and advantages facing S&IS and how these may direct and indirect effects on health financing, health system performance and health outcomes. Section 5 gives the results of the cross-country descriptive and comparative analysis of S&IS health financing status and progress towards UHC. Section 6 concludes with a brief discussion.

## 2 Definition of small and island states

Srinivasan (1986) notes to classify countries as ‘small’ two things are required; a measure of size and a threshold of this measure below which countries are defined as small. While there is debate on the relevant measure(s), absolute population is most commonly used.^2^ However, population thresholds used for a country to be considered ‘small’ have varied considerably – population thresholds of 1 million (Easterly and Kraay, 2000); 1.5 million (Azzopardi-Muscat and Camilleri, 2018; the World Bank Small States Forum (SSF); the Commonwealth; the IMF) 3; 2 million (Domerland and Sander, 2007, Favaro, 2008); 3 million (Armstrong and Read, 1998, Armstrong et al., 1998, Azzopardi-Muscat et al., 2016); 4 million (Winters and Martin, 2004); 5 million (Chenery and Syrquin, 1975, Collier and Dollar, 1999, Bräutigam and Woolcock, 2001); 10 million (Kuznets, 1960; Streenten, 1993) have been used.

As noted, there are many different initiatives defining countries which fall into this category ranging from approximately 30 to > 50 countries (Appendix A). Countries included in these lists are very heterogeneous.^4^

In this paper we focus on African S&IS, specifically the Africa region countries constituting members of the World Bank SSF, the same countries studied by Domëland and Sander (2007) (Table 1). These fourteen countries, represent 26 % of all African countries but only 1.53 % of its total population (United Nations World Population Prospects, 2022).^5^

**Table 1:** African countries classified as S&IS.

**AU Small and Island States**							
**Country**	**Population (2022)**	**Population ordinal global ranking**	**Land Area (sq. km)**	**Population density (per km sq.)**	**GDP Per Capita (2021 US$)**	**GDP Per Capita (2021 Int$ - PPP-adjusted)**	**Income Classification (2021)**
Botswana	2607,583	142/195	566,730	5	6805	16,449	UM (3)
Cabo Verde	590,503	167/195	4030	147	3293	7275	LM (2)
Comoros	829,245	160/195	1861	446	1577	3563	LM (2)
Djibouti	1113,147	158/195	23,180	48	3150	5421	LM (2)
Eswatini	1197,454	157/195	17,200	70	3978	9773	LM (2)
Equatorial Guinea	1655,207	150/195	28,050	59	7507	16,151	UM (3)
Gabon	2365,207	144/195	257,670	9	8635	15,244	UM (3)
Gambia	2672,890	141/195	10,120	264	772	2291	L (1)
Guinea-Bissau	2083,089	148/195	28,120	74	795	2021	L (1)
Lesotho	2294,313	145/195	30,360	76	1094	2530	LM (2)
Mauritius	1299,134	155/195	2030	640	9106	23,064	UM (3)
Namibia	2549,182	143/195	823,290	3	4866	10,161	UM (3)
Sao Tome and Principe	225,155	176/195	960	235	2361	4471	LM (2)
Seychelles	106,887	182/195	460	232	14,653	30,503	H (4)
**African S&IS Mean**	1542,071	-	128,147	165	4900	10,637	2.4
**African non-S&IS Mean**	31,623,366	-	696,460	93	1726	4698	1.5
**African Mean (unweighted)**	25,614,028	-	549,120	112	2580	6297	1.7

**Notes:** Population estimates and ranking from the UN World Population Prospects 2022. Country land size and GDP per capita from World Bank World Development Indicators. World Bank Analytical Country Classifications use GNI per capita. Data from Bank FY23 corresponding to 2021 calendar year.

We focus on a regional sub-group of S&IS, rather than the often-adopted global focus of research on S&IS which has been critiqued. As Voyatzis-Bouillard and Kelman (2021) notes these special interest groupings are more political, and therefore of less value for analytical purposes. Taking a regional focus removes some of the geographical heterogeneity and enables a move away from some broad generalisations, many of which do not hold for all countries to which they are applied. Additionally, Africa’s population is expected to grow by approximately 75 % between 2022 and 2050, reaching 2.4 billion. Although the proportion of the total African population in African S&IS will shrink from 1.53 % (2022) to 1.29 % (2050), these countries will become home to 32 million people by 2050. Therefore, without acknowledging the distinct circumstances and idiosyncratic challenges facing their health sectors, this population of 32 million people risks being overlooked by their larger faster growing neighbours in attempts to achieve UHC.

## 3 Methods

We undertake an assessment of the health financing status, health systems and unique challenges and opportunity which affect progress towards UHC of African S&IS using two approaches. First, we undertook a narrative review of the published academic and grey literature exploring health systems in S&IS. This involved a literature search performed using bibliographic databases (Econlit, PubMed and Google Scholar) to identify research articles examining health systems in S&IS.

The search was centred on literature focusing either on theoretical challenges or empirical studies related to health system issues in the 14 African S&IS. However, some exceptions were made for studies exploring wider LMIC S&IS where deemed relevant. Further, a snowballing strategy was used by reviewing the reference lists of identified studies to uncover further relevant literature. We group the studies using the WHO building blocks framework to build a picture of the current status of health systems in African S&IS and the idiosyncratic issues they face.

Second, we provide a descriptive cross-country comparative analysis of health system features of African S&IS, situating them within the wider African context. Specifically, we focus on three dimensions: i) health outcomes ii) health financing, and iii) health system inputs. Given the various data sources, not all data refers to identical time periods, nor is expressed in the same currency terms. All periods and value terms are made explicit in each case.

### 3.1 Data

#### 3.1.1 Health financing data

Data on GDP is from the World Bank World Development Indicators (WDI) for the period 2010–2021 expressed in real terms in constant US$2015. For tracking health expenditure, we use WHO Global Health Expenditure Data (GHED), the most commonly cited international mechanism for monitoring and benchmarking health spending across countries and over time. The GHED provides comparable health expenditure estimates for 190 countries since the year 2000 following the System of Health Accounts 2011 (SHA, 2011) framework. We use GHED covering health expenditure from 2010 to 2020, which includes the first year of the COVID pandemic expressed in constant US$2020 (WHO, 2022).^6^

#### 3.1.2 Health system inputs and performance data

Similarly, several data sources have been used to describe supply-side factors of healthcare systems. We use information on health infrastructure captured by Maina et al. (2019), containing a list of 98,745 geo-coded public and not-for-profit health facilities in 50 countries in sub-Saharan Africa.^7^ We also capture information on hospital beds and health workers using data from the WHO Global Health Observatory (GHO), with most data covering the period 2017–2021.

Data from the Global Burden of Disease, Injuries, and Risk Factors Study (GBD) 2019 is used to examine health systems performance in achieving Effective UHC coverage and indications of the quality of health care systems. The GBD UHC Effective Coverage index assesses the performance of health systems in delivering essential health care services. The index goes beyond measuring coverage of services, also capturing whether health systems deliver services aligned with countries health needs, and of sufficient quality to improve health outcomes (Ng et al., 2014). The index focuses on 23 essential interventions (Lozano et al., 2020). The index is based on country-specific disease profiles, enabling cross-country comparability, based on whether countries are maximising effective coverage for their specific context. Higher overall scores suggest better country performance at increasing access, better aligning essential service provision to the prevailing disease profile and health needs, and increasing the quality of essential health care services.

Additionally, we use data on the GBD Health Access and Quality (HAQ) index. While the UHC Effective Coverage index focuses on the delivery of essential health services within health systems, the HAQ examines broader health system performance as measured by its ability to reduce amenable mortality. The index risk-standardises mortality rates, removing the influence of behavioural and environmental risk factors, such that it measures only the effect of health system performance (Haakenstad, 2022). The index focuses on 32 causes of amenable mortality based on Nolte, McKee (2004), to measure health care access and quality both over time and between countries. The study also enables exploration of how health systems are performing for reducing amenable mortality for different age demographics.

#### 3.1.3 Health outcomes

World Bank World Development Indicators (WDI) are used to compare various health outcomes between African S&IS and non-S&IS. Specifically, we examine life expectancy, maternal and neonatal mortality rates as well as the prevalence of the most high-profile communicable diseases (HIV/AIDs, Malaria, Tuberculosis), and the relative importance of NCDs.

## 4 Narrative review of health financing and systems in African small and island states

Despite the longstanding categorisation and recognition of the challenges facing S&IS, until recently health has not been highlighted as a priority issue. In 2021 the WHO launched the Small Island Developing States (SIDS) Initiative at the SIDS Summit for Health (World Health Organisation, 2021). This represented the first global meeting focused exclusively on the health challenges facing SIDS. In 2023, technical and ministerial meetings were held with a focus on non-communicable diseases (NCDs) and mental health in SIDS (World Health Organsiation, 2023).

A small literature has examined health systems in S&IS. We have grouped previous literature examining health systems in S&IS using the WHO building blocks (World Health Organization, 2007).

### 4.1 Epidemiology and service delivery

Directly exploring whether size matters for health-related outcomes, Azzopardi-Muscat and Camilleri (2018) examine the relationship between population size and health outcomes measured by life expectancy, health resources measured by health care expenditure per capita and determinants of health represented by body mass index (BMI). They find, among LMICs, countries with smaller populations have higher life expectancies, while among HICs the opposite holds. However, this result appears largely driven by small states having the highest GDP per capita among the LMIC sub-group and lowest GDP per capita among the HIC sub-group. Similarly, among LMICs, they find smaller states have higher health expenditure per capita, while among HICs, health expenditure per capita is lower in smaller states. Again, the uncontrolled confounding factor of GDP per capita within these groups likely drives these identified associations. Finally, among LMICs a negative relationship is identified between population size and BMI. However, overall, richer countries are associated with higher average BMI, therefore, this could once more reflect small states having higher GDP per capita among LMICs. Additionally, among LMICs, BMI is unlikely to be a good signal reflecting determinants of health. Overall, the authors note that any explanatory power of population size disappears when GDP per capita is controlled for.

A small number of studies have examined the epidemiological and disease profile of S&IS. Theodore et al. (2011) highlight the impact of HIV/AIDS in small states, making the case that the economic disruption caused by HIV/AIDS epidemics is exacerbated. HIV/AIDS has long been recognised as a challenge to economic development as well as a health issue (Haacker, 2002). Several idiosyncratic features of S&IS mean they are even more economically and socially vulnerable, resulting in a potentially more severe impact. Unlike many other diseases, HIV/AIDS predominantly impacts working-age populations (15–49 years). As such, HIV/AIDS can reduce the size and productivity of a country’s labour force. The economic impacts of this are more severe in S&IS where the already smaller pool of labour means impacts on labour supply is more acutely felt, and demographic and population structures are more affected. As well as destroying human capital through its mortality and morbidity effect, it can also impact investment in and the accumulation of human capital by i) reducing the returns to investment in human capital; and ii) increasing the number of orphans in a country (Haacker, 2004). Considering many S&IS are relatively resource-limited and therefore more reliant on human capital, this effect can be economically significant. Theodore et al. (2011) note that four S&IS in Southern Africa (Botswana, Lesotho, Namibia and Eswatini) faced incredibly high HIV prevalence rates. They estimate that between 2001 and 2021, Botswana’s economy was 25 % smaller than it would have been without the HIV/AIDS epidemic.

Recognising the difficulties of providing specialised health care services, Suzana et al. (2018) examine overseas medical travel in S&IS. They identify significant expenditure growth on medical travel which rose from $0.68 million to $3.11 million between 2003 and 2013 in a sub-set of S&IS. This was largely driven by increased health-related travel expenses by the Maldives, Tuvalu, and Seychelles, which operate publicly funded overseas medical travel schemes. The authors suggest government subsidised medical travel schemes might assist in achieving UHC in S&IS where investments in specialist health care may not be economically feasible. However, they highlight the possible cost and equity issues such schemes can raise. Similarly, Mauritius has a policy of financial assistance for overseas treatment for households earning less than MUR100,000 (US$2000) per month (Government of Mauritius, 2022).

### 4.2 Health financing

Size can also influence health financing arrangements. Smith and Witter (2004) note the importance of population size for health insurance and risk pooling performance. Risk pooling is vital for preventing individuals from bearing the full financial cost of utilising health care, thereby improving financial risk protection (FRP), a key component of UHC. The level of uncertainty in predicting expenditure decreases as risk pools grow, as random variation in health care needs reduces in importance. Therefore, small populations may limit the ability of social or private health insurance schemes (such as local government or employer-based pools) to efficiently operate, as, all else equal, larger risk pools reduce premiums.^8^ Additionally, it may reduce the ability to devolve health budgets to sub-national levels (Martin et al., 1998). Finally, the size of risk pools may influence the type of health interventions covered, as smaller risk pools may be appropriate for more predictable routine care, but not for more expensive less common interventions where random variations in need can have a significant financial impact.

African S&IS appear to have made good progress on FRP. The WHO (2022) suggests between 2000 and 2019, only six WHO African Region member states have managed to increase health service coverage while simultaneously reducing the incidence of catastrophic health expenditure (CHE), of which four are African S&IS (Algeria, Cabo Verde, Mauritius, Namibia, Seychelles and South Africa).

Relatively few studies on FRP have taken place in S&IS. In a scoping review of studies on FRP from out-of-pocket health spending in LMICs Rahman et al. (2022) identified only one study focused on an African S&IS (of 155 studies included in the review). In this study, Ngcamphalala and Ataguba (2018) assess the incidence of CHE in Eswatini. Using a threshold of 10 % of household income, 9.7 % of households suffered CHE in 2009/10. Nundoochan (2021) explore how equitable health financing is in Mauritius, looking at which socioeconomic groups pay for and benefit from health care. The distribution of health care benefits was found to be pro-poor in the public sector, although the degree of this was reduced when accounting for differences in need. However, the private sector was pro-rich, resulting in an overall (combined public and private) regressive distribution of benefits when accounting for need. Additionally, the health financing system is found to be regressive. Whether country size impacts the ability of countries to mobilise domestic resources to finance progressive health systems remains an open question.

In a similar review, Eze et al. (2022) identify the following 3 studies (of 89 total) examining FRP in African S&IS. Akinkugbe et al. (2012) find the proportion of households facing CHE using a 40 % threshold was 7 % and 1.25 % for Botswana and Lesotho respectively in 2002/3. Nundoochan et al. (2019) found the incidence of CHE in Mauritius rose from 0.61 % in 2001/2–1.25 % in 2012 using the same threshold. Using World Health Surveys (WHS) 2002/3, Saksena et al. (2010) examine the drivers of CHE in 51 countries including Comoros, Namibia, Mauritius and Eswatini. In the 2019 Global Monitoring Report on Financial Protection in Health, 56 % of the countries without data are S&IS (WHO/World Bank, 2019).

Additionally, the disproportionate expenditure on tertiary care in S&IS is frequently cited as an issue. Asante et al. (2017) note that > 70 % of government expenditure on health care is on hospital services in Fiji.

### 4.3 Health workforce

Several studies have examined brain drain in S&IS. De la Croix et al. (2013) examine emigration in SIDS, finding rates to be far above larger developing countries.^9^ They identify a strong relationship between country size and emigration rates, affecting the capacity of SIDS to accumulate human capital. Medical brain drain (MBD) in S&IS can be problematic due to low absolute numbers of health workers. Bhargava et al. (2011) find 12 of the 30 countries with the highest rates of physician brain drain in 2004 were UN SIDS. However, no African S&IS – as defined in this paper – are among the top 30, while 9 large African countries are. Building on this work, Adovora et al. (2021) find 11 of the 20 countries with the highest rates of physician brain drain in 2014 were UN SIDS. However, again no African S&IS are in this list, while 5 larger African states are. Overall physician migration rates from sub-Saharan Africa have slightly decreased between 2004 and 14. However, they find the average MBD in small countries (defined as <2.5 million) was 34 % in 2014, compared to 3.9 % in countries with populations greater than 25 million. Among the country groupings examined, small countries saw the highest increase in MBD rates, from 9.9 % to 34 % between 1991 and 2014.

### 4.4 Medical products, vaccines and technologies

S&IS face unique disadvantages in global medicine markets. First, relatively smaller market size and demand may result in lower prioritisation or negotiating power compared to larger countries placing higher volume orders. However, Garuoliene et al. (2014) find that Lithuania is able to negotiate similar generic price discounts to larger European peers, suggesting S&IS are not always disadvantaged regarding price negotiations. Second, S&IS often face logistical challenges due to complex or isolated geographies. Together, these issues can lead to higher costs and greater unpredictability in the supply of medicines. S&IS small domestic markets also prohibit the development of local manufacturing, resulting in a high exposure to supply chain disruptions leading to medicine shortages and stock outs. However, there is little research on medicine pricing policies in S&IS. In a systematic review Koduah et al. (2022) identify medicine pricing policies in 22/46 sub-Saharan African countries, with only four S&IS. Rewari et al. (2020) note that reduced air freight capacity due to COVID increased transportation costs led to pharmaceutical manufacturers being less likely to accept small-volume orders. This problem is equally applicable to medicines for neglected tropical diseases (NTDs) or rare conditions which have small markets. Stakeholders estimated a 2–6 times increase in the pre-pandemic cost of freight (World Health Organisation, 2023).

Further, noting the trade-off between more stable multi-source procurement processes and single source procurement which benefits from economies of scale but is more susceptible to disruption, World Health Organisation (2023) suggests the former option may not be viable in small markets, highlighting this as further rationale for pooled procurement processes across countries. This is more challenging for the geographically dispersed African S&IS compared to the Caribbean. Many examples of pooled purchasing arrangements exist, such as the Pan American Health Organisation (PAHO) EPI Revolving Fund (DeRoeck et al., 2006). WHO Africa Region SIDS discussed the possibility of pooled procurement in 2019.^10^ Separately, the African Continental Free Area (AfCFTA) Pharmaceutical Initiative was established in 2019 to facilitate i) localised production of medicines ii) pooled procurement iii) harmonised regulatory and quality framework.^11^ The Initiative’s Centralised Pooled Procurement Mechanism was established in 2021. However, Parmaksiz et al. (2022) highlight several attempts to establish pooled procurement initiatives which ultimately failed, even after early stage implementation.

### 4.5 Leadership/governance/health information

Meessen et al. (2024) note that the effectiveness or even ability to implement many health system strengthening (HSS) policies and programmes may be related to the size of a health system, and the state. Certain policies may impose high fixed costs or require complementary inputs which impose high burdens on countries with smaller administrations. Therefore, HSS policies benefiting from returns to scale may need to be adapted to be effective in smaller states. Meessen et al. (2024) also note that SIDS have been slow in developing modern health information systems (HIS). In 2016, 60 % of pacific islands reported not having health information system policies or legislation (World Health Organization, 2017). However, S&IS may have an easier time implementing strong HIS due to the smaller number of facilities and closer links to central support. Eswatini is in the process of implementing one of the more advanced patient-based electronic health records system with its Client Management Information System (Measure Evaluation, 2017).

## 5 Cross-country comparative analysis

### 5.1 Health outputs and outcomes

Table 2 shows the differences in health outcomes between African S&IS and non-S&IS. Despite greater per capita expenditure on health and higher levels of effective UHC (Table 3 below) in non-S&IS, life expectancy among African S&IS and non-S&IS is similar. Although African S&IS on average perform relatively better for MMR and U5MR, there is significant heterogeneity with two S&IS - Guinea-Bissau and Lesotho - among the worst performing countries globally for MMR. The HIV prevalence illustrates the problem infectious diseases can cause in smaller populations, as noted by Theodore et al. (2011). Botswana, Lesotho and Eswatini have the highest 10-year average HIV prevalence rates in the AU, with 21.3 %, 23.6 % and 28.9 % respectively. Next highest are South Africa (17.9 %), Zimbabwe (13.8 %), Namibia (12.9 %) and Zambia (12.3 %). Even excluding the top 3 countries, the African S&IS average is 3.3 % which is higher than the African non-S&IS average of 2.9 %. Similarly, 5 of the top 10 African countries with the highest average annual TB incidence rate are African S&IS. However, two of the five countries in Africa to achieve the 95–95–95 HIV targets in 2022 were S&IS (Botswana and eSwatini) (World Health Organisation, 2023).

**Table 2:** Health outcomes.

**Country**	**Life Expectancy**	**Maternal Mortality Rate (MMR)**	**Under-5 Mortality Rate (U5MR)**	**HIV Prevalence**	**TB Incidence (per 100,000 people)**	**Malaria Incidence (per 1000 population at risk)**	**% deaths from Non-communicable Disease**
Botswana	63.3	168.6	46.2	21.3	351.4	0.8	42.8
Cabo Verde	75.0	45.7	19.5	0.6	64.6	0.3	66.0
Comoros	62.9	267.1	60.5	0.1	34.6	26.0	41.8
Djibouti	61.9	250.8	65.0	1.2	361.8	27.0	46.0
Eswatini	54.6	397.5	65.5	28.9	755.5	1.2	42.1
Equatorial Guinea	60.0	200.5	92.8	6.5	260.8	265.2	32.1
Gabon	65.4	208.7	49.9	3.7	535.4	236.9	44.4
Gambia	62.4	537.4	59.7	1.8	168.0	145.0	35.0
Guinea-Bissau	59.0	724.5	91.8	3.6	361.0	99.0	31.0
Lesotho	50.9	771.7	81.4	23.6	820.2	0.0	42.6
Mauritius	74.0	52.8	15.2	1.5	12.3	0.0	87.4
Namibia	60.1	302.5	45.3	12.9	630.9	12.4	42.0
Sao Tome and Principe	67.0	153.5	23.7	0.8	127.5	22.1	53.7
Seychelles	73.9	5.4	14.4	0.0	17.5	0.0	78.2
**African S&IS Mean**	62.4	333.3	60.6	8.2	321.5	76.0	48.9
**African non-S&IS Mean**	62.2	435.9	70.5	2.8	213.8	210.1	40.0
**African Mean**	62.3	408.8	67.8	4.2	242.3	179.4	42.4

**Notes:** The average of annual values over the period 2010–2021 is reported, except for % of deaths from NCDs for which data in 2015, 2017 and 2019 is available. World Bank World Development Indicators (WDI).

In addition to infectious diseases, small populations can also exacerbate other health issues. Seychelles and Mauritius have the highest rates of heroin use per capita globally. This was estimated at 10 % of Seychelles national workforce in 2019 (5 % of the total population) (Bird et al., 2021).

However, African S&IS have relatively lower rates of Malaria. Seven countries have been declared Malaria free by the WHO.^12^ However, in sub-Saharan African, only three countries, all S&IS, (Lesotho, Mauritius, Seychelles), have achieved this status. Further, five of the six African countries which are part of the WHO programme targeting malaria elimination by 2025 (E-2025) are S&IS (Botswana, Cabe Verde, Comoros, Eswatini, Sao Tome & Principe, South Africa). Therefore, in addition to providing unique challenges, S&IS also provide a unique opportunity to achieve disease elimination.

### 5.2 Health financing

**Fig. 1:**
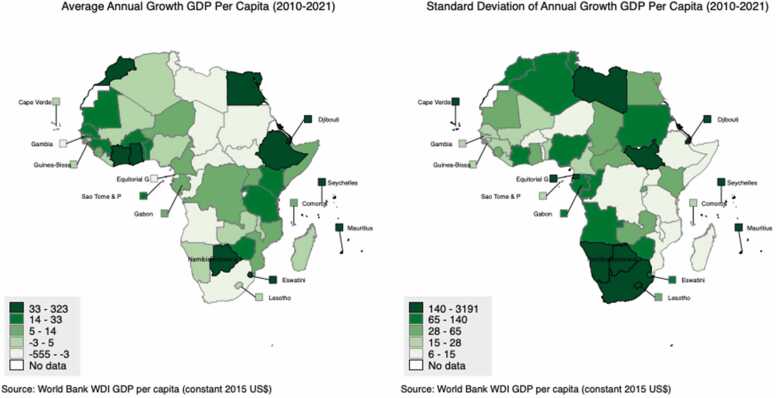
GDP Growth and Variation in AU Countries 2010–21. Source: Authors calculations based on WDI data.

As health financing is influenced by countries general economic health, we briefly examine recent trends in relative GDP growth among S&IS. As illustrated in Table 1 the average GDP per capita of African non-S&IS is 35 % that of African S&IS (44 % PPP-adjusted). Fig. 1 illustrates recent economic growth and volatility in Africa. Fig. 1(a) shows countries average annual GDP per capita growth from 2010 to 21 while Fig. 1(b) is the standard deviation of countries annual growth in GDP per capita over the same period. Although the average annual GDP per capita growth is higher in African S&IS (US$30) compared to African non-S&IS (US-$8), S&IS are relatively uniformly split across quintiles of average annual per capita growth (6 S&IS are in the top two quintiles and 6 in the bottom two quintiles). However, when looking at volatility in growth, as measured by the standard deviation of annual GDP per capita growth, 70 % of the top quintile are S&IS (i.e., half of African S&IS are in the top quintile). Further, no S&IS are in the bottom quintile. This echo’s the findings of Easterly and Kraay (2000), that African S&IS do not have substantively different per capita growth rates than larger African states but there does appear to be greater volatility in the annual growth rates, possibly reflecting the greater exposure to shocks.

**Fig. 2:**
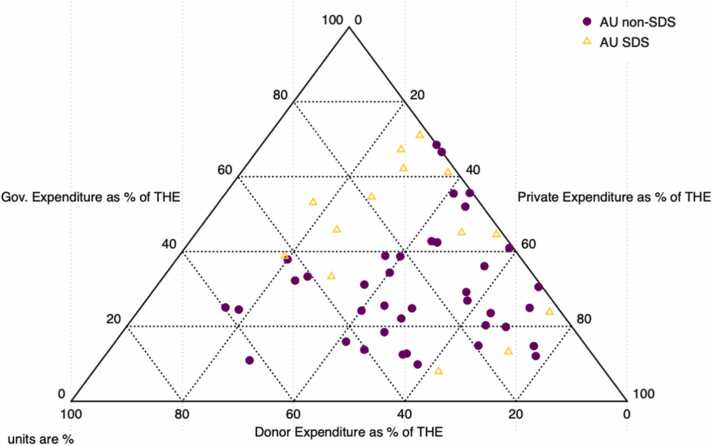
Sources of Health Expenditure in African Union States (average 2010–2020). Source: Authors calculations based on GHED data.

Table 3 provides a high-level comparison of health financing in African S&IS and African non-S&IS. Average figures over an eleven-year period (2010–2020) are reported, rather than the latest year for which data is available. This gives a medium- to long-term indication of expenditure patterns. Column A confirms previous findings that public spending as a percentage of GDP is higher in small states (Alesina and Wacziarg, 1998, Rodrik, 1998, Favaro, 2008). Table 3 also makes clear that total health expenditure (THE) per capita is much higher among African S&IS, at almost 3 times the non-S&IS per capita spending. However, given that THE as a percentage of GDP are relatively comparable, this higher health spending per capita is partly related to the differences in average GDP per capita among S&IS and non-S&IS. Column D shows the average proportion of general government expenditure (GGE) spent on health is ∼21 % higher among African S&IS. This suggests a health and health care is given a higher prioritisation among African S&IS, which is consistent with the idea that human capital, of which health is a major determinant, is often more important for countries with small populations.^13^ However, no African country has met the Abuja Declaration target to allocate at least 15 % of annual government budget to health consistently over the period 2010–2020 (African Union, 2001). Although South Africa averaged 14.9 % of general government expenditure allocated to health over the period.

**Table 3:** Aggregate health care financing.

	**Column A**	**Column B**	**Column C**	**Column D**	**Column E**	**Column F**	**Column G**	**Column H**
**Country**	**General Government Expenditure (GGE) as % GDP (%)**	**Total Health Expenditure (THE) per Capita (US$)**	**THE as % GDP (%)**	**Government Health Expenditure as % GGE (%)**	**Government Health Expenditure as % THE (%)**	**Private Health Expenditure as % THE (%)**	**Donor Health Expenditure as % THE (%)**	**Out of Pocket Payments (OOPP) as % THE (%)**
**Botswana**	37	399	6.2	11.2	67.3	25.7	7.0	4.1
**Cabo Verde**	33	164	5.1	9.6	62.2	28.7	9.2	26.2
**Comoros**	17	75	5.1	4.1	13.3	72.0	14.7	68.4
**Djibouti**	28	64	2.7	5.5	54.6	26.7	18.7	25.4
**Eswatini**	32	288	7.4	10.7	45.8	24.9	29.3	10.6
**Equatorial Guinea**	27	274	2.5	2.4	23.8	74.1	2.1	70.6
**Gabon**	22	221	2.8	7.9	61.1	37.2	1.7	25.3
**Gambia**	20	24	3.5	5.8	33.3	30.1	36.5	22.8
**Guinea-Bissau**	19	52	7.6	3.2	8.0	62.1	29.9	58.6
**Lesotho**	55	109	9.8	9.4	53.1	17.0	29.9	16.3
**Mauritius**	26	513	5.4	9.4	44.5	54.2	1.3	49.2
**Namibia**	39	481	9.1	10.6	45.1	47.7	7.2	8.7
**São Tomé and Principe**	34	102	6.3	7.7	38.8	19.0	42.2	17.1
**Seychelles**	37	668	5.0	9.6	71.1	27.2	1.8	25.6
**African S&IS Mean**	30	245	5.6	7.6	44.4	39.0	16.5	30.6
**African Non-S&IS Mean**	24	86	5.2	6.3	30	46.9	23.2	39.7
**African Mean**	26	128	5.3	6.7	34	44.8	21.5	37.3
**S&IS Countries**	14	14	14	14	14	14	14	14
**S&IS Sample**	154/154	154/154	154/154	154/154	154/154	154/154	154/154	154/154
**Non-S&IS Countries**	39	39	39	39	39	39	39	39
**Non-S&IS Sample**	413/429	413/429	413/429	413/429	413/429	413/429	413/429	413/429

**Notes:** GHED data. Statistics represent 11-year average from 2010 to 2020. US$ expressed in real terms In constant US$2020. The difference between private expenditure and OOPP reflects, among other things, privately purchased health insurance schemes.

**Fig. 3:**
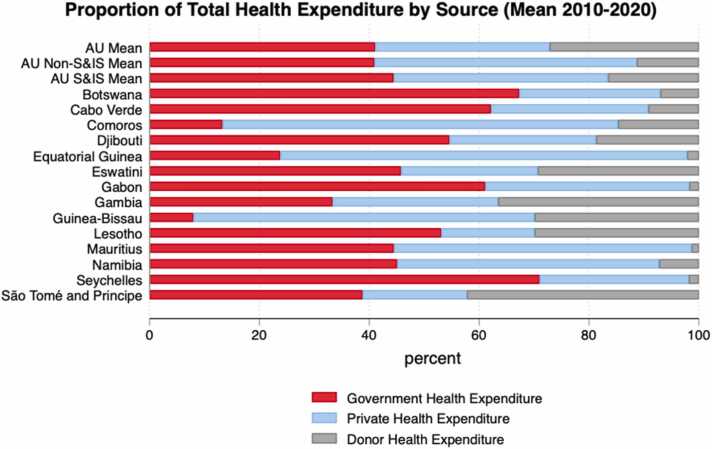
Composition of THE. Source: Authors calculations based on GHED data.

Fig. 2, Fig. 3 show the composition of health expenditure among African countries. While there is a high degree of heterogeneity it is clear that government spending makes up a substantially higher proportion of THE among S&IS relative to their larger peers, while donor expenditure constitutes a smaller share of their THE (Column D-H Table 3, Fig. 2).

Finally, column H shows the average proportion of OOP payments for African S&IS is 23 % lower than non-S&IS. This lower OOP payments are resulting in lower CHE in African S&IS. The latest evidence suggests, on average, 4.24 % and 0.93 % of African S&IS households are spending greater than 10 % and 25 % of income on health, compared to 7.91 % and 1.86 % in African non-S&IS (see Appendix B). Combined with the information on access and utilisation of health care (below), this suggests S&IS better achieve goals related to financial risk protection from health care-related expenditures.

**Fig. 4:**
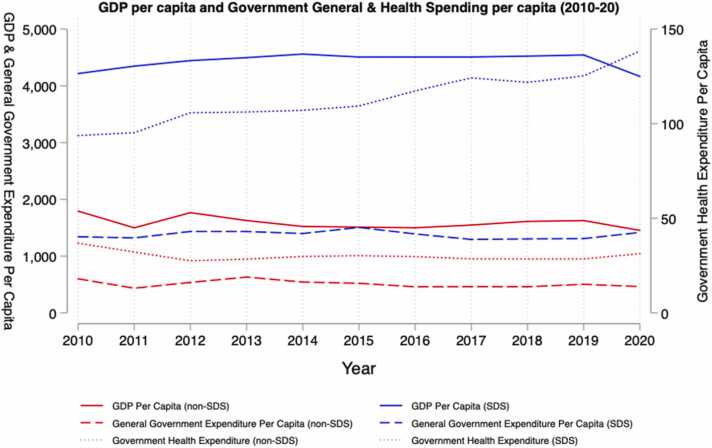
Time Series of GDP, GGE and Government Health Spending Per Capita. Source: Authors calculations based on GHED data.

Fig. 4 shows the evolution of GDP per capita, general government expenditure (GGE) per capita and government health expenditure (GHE) per capita from 2010 to 21. Average GDP per capita has fallen slightly from both groups from 2010 to 20. GGE per capita has fallen from US$600 (2010) to US$464 (2020) for non-S&IS, while it has risen from US$1341 (2010) to US$1417 (2020) in S&IS. Similarly, GHE per capita has slightly fallen in non-S&IS from US$37 (2010) to US$31 (2020), while it has risen in S&IS from US$94 (2010) to US$138 (2020). Therefore, GHE per capita fell by 17 % in non-S&IS while it increased by 47 % in S&IS between 2010 and 2020. Despite this, the proportion of government spending per capita spent on health in both non-S&IS and S&IS increased over this period, from 6.2 % to 6.7 % and from 7 % to 9.7 % in non-S&IS and S&IS respectively. However, for non-S&IS, it would be generous to paint this increase in the proportion of government spending going towards health as a sign of an increasing prioritisation towards health spending. While for S&IS it seems clear that health spending is being increasingly prioritised.

### 5.3 Health infrastructure and health workforce

Table 4, Table 5 show that the per capita supply of health infrastructure and health care workers are much higher in African S&IS. Table 4 shows that S&IS have almost double the hospitals per capita, more non-hospital health infrastructure and substantially more bed capacity than African non-S&IS.^14^ Combined with the smaller land areas and higher population density in most African S&IS (Table 1), this suggests populations in S&IS may have better geographical accessibility to health care. Table 5 shows a similar pattern for health workers, with S&IS benefiting from more doctors, nurses and midwives, and community health workers (CHWs) per capita than larger African countries. Given CHWs arguably lower value in contexts with smaller distances to access health infrastructure, we would expect a lower CHW presence.^15^ However, the average number of CHWs per capita in S&IS is significantly influenced by Eswatini and Lesotho, which have the highest number of CHWs per capita in among African all countries (only Rwanda and Uganda have >40 CHWs per 10,000). Overall, only three S&IS have more than the 9.2 CHWs per 10,000 average of non-S&IS. Given Botswana and Namibia’s large geography, we might expect higher CHW per capita rates than displayed. However, CHW numbers also reflect differences in national health policy decisions. Overall, while Adovora et al. (2021) found that while medical brain drain may be an issue for UN SIDS and small countries generally, Table 4 shows African S&IS are in a more favourable positions compared to larger African countries, for whom this poses a greater health policy issue.

**Table 4:** Heath infrastructure.

**Country**	**Hospitals per 10,000**	**Other health infrastructure per 10,000**	**Total health infrastructure per 10,000**	**Hospital Beds per 10,000**
Botswana	0.12	2.45	2.57	18
Cape Verde	0.16	1.00	1.16	21
Comoros	0.04	0.82	0.86	22
Djibouti	0.12	0.51	0.63	-
Eswatini	0.05	1.12	1.17	21
Equatorial Guinea	0.12	0.20	0.32	21
Gabon	0.07	2.43	2.50	13
Gambia	0.02	0.40	0.43	11
Guinea Bissau	0.04	-	0.04	10
Lesotho	0.09	0.44	0.54	13
Mauritius	0.08	1.20	1.28	34
Namibia	0.15	1.40	1.55	27
Sao Tome and Principe	0.10	2.29	2.38	29
Seychelles	0.10	1.66	1.76	36
**AU S&IS Mean**	0.09	1.14	1.23	21
**AU non-S&IS Mean**	0.05	0.85	0.89	9
**AU Mean**	0.06	0.93	0.99	12
**AU S&IS Average Year Data**	-	-	-	2009.8
**AU non-S&IS Average Year Data**	-	-	-	2009.6
**AU Average Year Data**	-	-	-	2009.7

**Notes:** Data on health infrastructure only relates to sub-Saharan Africa i.e. means calculated only using countries from this region. Data on Hospital Beds includes 46 countries across whole AU region. Data on lower tier facilities for Guinea Bissau could not be located (Maina et al., 2019). Data on hospital beds in Djibouti is not captured (WHO GHO).

**Table 5:** Heath workers.

**Country**	**Doctors per 10,000**	**Nurses & Midwives per 10,000**	**Community Health Workers per 10,000**
Botswana	3.5	50.2	3.0
Cabo Verde	7.9	12.4	2.1
Comoros	2.8	15.9	-
Djibouti	2.0	6.6	-
Eswatini	1.4	24.7	45.9
Equatorial Guinea	3.5	2.7	3.8
Gabon	5.9	26.8	0.5
Gambia	0.8	8.9	8.2
Guinea-Bissau	2.2	10.5	18.6
Lesotho	4.5	31.2	66.4
Mauritius	26.6	38.5	1.3
Namibia	6.0	19.9	8.5
Sao Tome and Principe	4.9	21.5	9.6
Seychelles	21.1	92.2	-
**AU S&IS Mean**	6.7	25.9	15.3
**AU non-S&IS Mean**	3.0	12.1	9.2
**AU Mean**	3.9	15.7	10.7
**AU S&IS Average Year Data**	2018.6	2018.4	2016.5
**AU non-S&IS Average Year Data**	2019.1	2018.8	2019.1
**AU Average Year Data**	2018.9	2018.7	2018.4

**Notes:** All data on Doctors is from 2017 to 2021, except Djibouti and Somalia for which the latest year is 2014. Means constructed with 54 countries for Doctors and Nurses & Midwives and 44 countries for Community Health Workers.

**Fig. 5:**
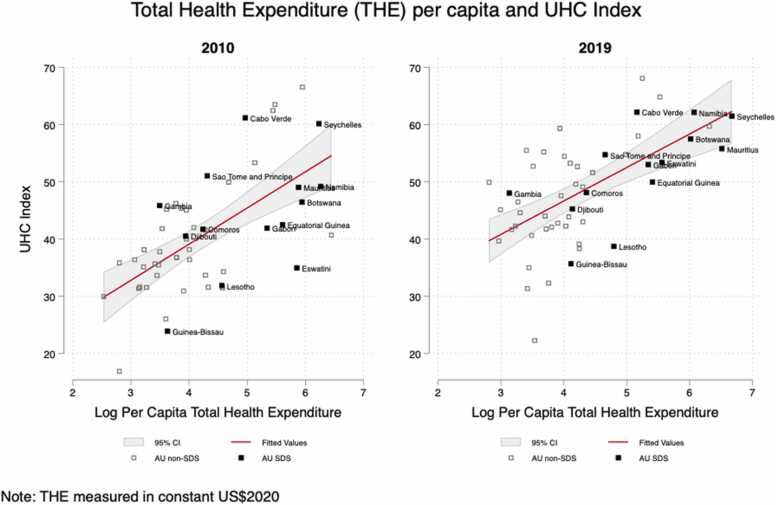
Relationship between THE and GBD UHC Effective Coverage Index. Source: Authors calculations based on GBD and GHED data.

Fig. 5 illustrates countries progress towards achieving UHC, as measured by the GBD UHC Effective Coverage Index. We use bivariate linear regressions to show the relationship between THE per capita and UHC effective coverage (see also Appendix C). There is a clear positive relationship between THE per capita and UHC effective coverage score. However, many African S&IS, despite high relative health expenditure per capita, are achieving lower UHC effective coverage scores than might be expected for their per capita expenditure levels.

However, the relationship between per capita health expenditure and UHC effective coverage is not straight-forward. A number of S&IS increased their THE per capita between 2010 and 19, but this was not accompanied by large improvements in UHC. Conversely, several S&IS saw THE per capita fall while simultaneously improving their UHC effective coverage (Appendix D).

While the quality of health care is largely undocumented in many S&IS, the GBD Health Access and Quality (HAQ) Index, offers some indication of the performance of S&IS. Appendix E shows the HAQ Index among different age groups (young (ages 0–14 years), working (ages 15–64 years), and post-working (ages 65–74 years)). The average HAQ index is higher for all age groups for S&IS. However, between 1990 and 2019 non-S&IS improved their HAQ index for < 15 more than S&IS. As previously noted, the population of working age may have more significance for S&IS given their frequent reliance on human capital for economic growth. Therefore, the importance of ensuring high-quality health care for this population to maintain a productive labour force. While Eswatini and Comoros do better than the African non-S&IS in providing essential health care services, they do worse at impacting amenable mortality. This potentially suggests a sub-optimal quality of health care in these countries.

**Fig. 6:**
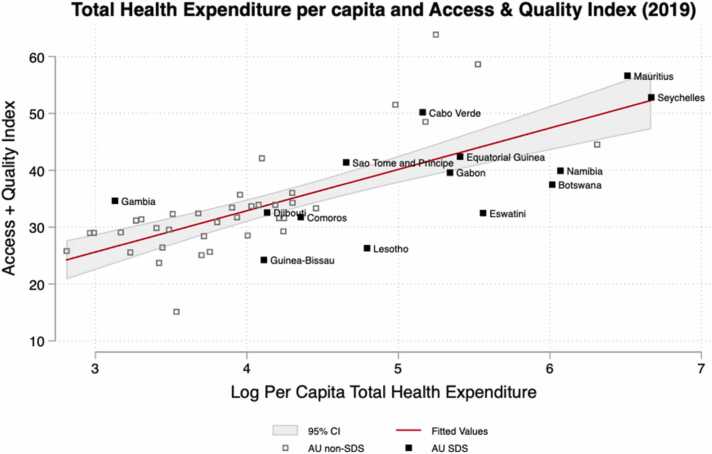
THE against GBD Access and Quality Index.

Fig. 6 makes clear that the higher average HAQ index among S&IS is related to higher spending. Many S&IS are achieving lower HAQ scores than they may be expected given their expenditure levels.

## 6 Discussion

In this paper, we synthesised the evidence on the specific challenges and advantages S&IS may face in financing and operating a sustainable health system towards achieving UHC. We also compared the status of African S&IS in health financing, supply-side factors such as health infrastructure and human resource for health, and health outcomes relative to their larger peers and interpreted these results informed by the findings and conclusions of previous literature.

While health systems vary across countries, African S&IS often face idiosyncratic issues and policy questions within health system pillars. Given their higher GDP per capita, African S&IS perform relatively well in health financing. Not only is there a higher THE per capita, but the prioritisation of health spending is higher in African S&IS. This may be due to S&IS governments recognising the outsized importance of human capital investment in determining their economic development (Briguglio, 2022). Exploratory evidence suggests African S&IS may also achieve higher FRP, with government expenditure constituting a higher proportion of THE. As noted by Piatti-Fünfkirchen et al. (2018), government spending on health reflects not just the degree prioritisation of health, but also the effectiveness of domestic resource mobilisation. This tends to increase with the level of economic development and accompanying formalisation of the economy. African S&IS may have a natural advantage in terms of resource mobilisation, beyond simply having a higher average GDP per capita. Given geographic characteristics, it is likely a lower proportion of African S&IS populations are involved in informal work (for example agriculture), potentially improving their ability to tax work and mobilise domestic resources (Brookings Institute, 2017).

Equally, African S&IS perform favourably when comparing key health system inputs such as infrastructure and health workers. One important distinction between African S&IS and their larger counterparts is their lower numbers of community health care workers. This likely reflects health policy choices, as geographic accessibility is less of a constraint in many S&IS and nurses and midwives may be used to perform similar outreach activities as CHWs in other settings. Given the higher per capita health expenditure of African S&IS, health system performance, as measured by the GBD UHC Effective Coverage Index and Access and Quality Index, is slightly higher.

However, there are several actions which should be undertaken – by both policy-makers and researchers – to gain a better understanding of the circumstances facing health systems in S&IS and continue progress towards UHC. First, all African region countries including African S&IS, must continue to increase the domestic resources which are dedicated to health. While the Abuja commitments represent an ambitious target, moving countries THE towards the average 7.5 % of GDP estimated to be required to achieve the SDG health targets represents a realistic goal for many African S&IS. Additionally, further research is required to explore if there are differences in the efficiencies of health expenditure in African S&IS and non-S&IS. Due to the challenges facing S&IS it is possible greater health expenditure is required to achieve similar health outcomes. For example, do economies of scale imply S&IS need to expend more resource per capita to achieve comparable aggregate outcomes as larger countries. In particular, Guinea-Bissau and Lesotho’s health systems appear to be underperforming given their level of health expenditure. The high MMR and U5MR seen in these countries suggest issues with the functionality of basic primary health care. As noted, S&IS often spend significant proportions of health financing on tertiary care. Given many S&IS are starting to see significant disease burdens from NCDs, this suggests the need for greater investments in preventative primary care. Many of the risk-factors related to the NCDs in S&IS are related to health behaviours, namely diet and exercise. Sin taxes have been increasingly proposed as a means of mobilising more resources for the health sector and improving health behaviours. For example, many countries are introducing sugar-sweetened beverages (SSB) taxes. To date, only one of the five East and Southern African S&IS (Mauritius) had implemented a sugar-content-based specific tax (Kadungure and Loewenson, 2023). This is despite evidence showing significant effects in reducing the consumption of SSB where they have been implemented (Pedraza et al., 2019). Given that S&IS, as a whole, have the highest rates of obesity globally, policies targeting the risk-factors should be considered health sector priorities.

If inefficiencies exist, these may be related to allocative or technical efficiency issues. S&IS health systems may be particularly vulnerable to the latter due to the inability to fully benefit from economies of scale and scope. As highlighted, this can manifest in higher costs for medical products and equipment, however, it can also impact the quality of health care. There is a hypothesised relationship between volume and quality of health care resulting from a ‘learning-by-doing’ effect (Ho, 2014). A number of studies have examined whether a relationship between volume and patient outcomes exists (Gaynor et al., 2005, Gutacker et al., 2017). Despite mixed evidence, some countries have set minimum volume regulation for certain health care interventions (Rachet-Jacquet et al., 2021). Given the quantity of health services delivered is related to population size, economies of scale issues have the potential to impact quality in S&IS. This suggests an urgent need for evidence on the quality of care in African S&IS. Very little is known about the quality of health care in S&IS, and while health outcomes look good on average relative to larger African countries, this may simply be due to greater health expenditure. If higher expenditure is simply off-setting lower technical (or allocative) efficiency, this would suggest once at similar levels of development, larger African countries, may be able to match and potentially better the health outcomes seen in African S&IS. Jarjue et al. (2015) find that most health facilities in the Gambia are both technically and scale inefficient. However, their findings are consistent with many other efficiency analyses undertaken across Africa, and do not provide evidence that S&IS are less efficient at the facility-level. However, this concern does suggest that in S&IS with low population densities, policy-makers may need to be mindful of balancing improved access with potential volume-related quality of care issues. This may suggest that an alternative to decentralising services to lower level providers, certain services may benefit from being consolidated at higher levels with a focus on strengthening referral networks.

Even if S&IS are found to suffer from structural inefficiencies, it is generally believed that smaller states may be more dynamic and find experimenting with policy reforms to overcome these easier (Handforth, 2020). Lesotho provides an example of an innovative approach where it engaged in a public-private partnership (PPP) for the co-financing, design, construction and operation of a new main tertiary hospital and three clinics in the capital (Hellowell, 2019). The policy was viewed as a flagship model for the continent, being Africa’s first and largest integrated PPP in health (McGuire et al., 2024). The scheme was agreed in 2009, with the hospital opening in 2011. However, in 2014 the hospital and clinics were consuming over 50 % of the total government health sector budget (Oxfam, 2014). The contract was originally scheduled to run until 2026, but due to the spiralling costs was cancelled in 2021. Limited state capacity in the ability to manage complex contracts was suggested as a factor for the problems incurred (Hellowell, 2019). This highlights the significant consequences that mis-judged health sector reforms can have for S&IS, and the need for S&IS to maintain a degree of caution with health sector policy reforms due to the increased potential of such catastrophic risks.

In addition to concerns around higher costs of medical products, over-reliance on external production may continue to be an issue**.** Several countries in Africa are investing to become pharmaceutical hubs (World Bank, 2021). It has been noted that this is particularly important given countries transition to middle-income status and the consequent loss of assistance from the Global Fund, GAVI and other donors supporting medicine procurement (U.S. Pharmacopeial, 2019). African S&IS will, as a result of their small domestic market size, struggle to develop local production of medicines, resulting in their continued almost total reliance on external sources of production. There is no empirical evidence on the relationship between local medicine production and the reliability of supply and access to medicines (WHO, 2011; Kaplan et al., 2011). However, this remains a vulnerability of S&IS health systems which will be difficult to address. Until further developments arise regarding domestic pharmaceutical manufacturing, pooled procurement initiatives (such as the current WHO African Region SIDS initiative) should continue to be pursued with accompanying political and financial support.

As noted, the issue of overseas travel for specialist medical treatment, the associated financial burden and continuity of care issues has been flagged as particularly problematic for S&IS (Suzana et al., 2018). In 2013, overseas treatment constituted 8.6 % and 18 % of THE in Sao Tome & Principe and Seychelles respectively. However, given the higher average THE of S&IS in Africa and that S&IS have higher doctors-to-population ratios, this possibly suggests that households in S&IS are travelling abroad for treatment which is unlikely to be available to populations even in larger African countries. Due to economies of scale and the transition to NCD, this issue is bound to grow more pronounced with escalating costs over time. Therefore, S&IS face a difficult policy-choice, invest in specialist care and accept higher average cost per patient for many specialist small volume services or publicly fund overseas medical care. The Government of Gambia is undertaking a joint venture with the private sector to construct a specialist hospital offering services not currently available in the country (African Development Bank, 2014). Primarily a private facility charging user fees, this may offer in-country referral opportunities as an alternative to overseas treatment. In 2017, Gambia’s full health budget was only sufficient to cover 17.6 % of its Basic Health Care Package (BHCP) (Government of Gambia, 2017b), despite the objective of Government financing at least 50 % of the BHCP (Government of Gambia, 2017a). This suggests the provision of specialist treatment may not currently be the most efficient use of resources. Such arrangements must be assessed on their costs and benefits from a health system perspective, requiring capacity in health economics and financing, an area where Gambia, partially due to its small size and the specialist nature of the topic is currently lacking (Jarjue et al., 2021). Given that many S&IS face the same policy choice, this is an opportunity for cross-country learning in identifying approaches and solutions. Linking to high-income S&IS who have faced this same choice can assist African S&IS better understanding the advantages and disadvantages of the different policy options. For example, the Islands and Small States Institute in Malta is a WHO Collaborating Centre on Health Systems and Policies in Small States, which could provide opportunities for sharing policy experiences.

Despite the studies highlighted in this paper, S&IS often remain overlooked, with most focus going towards their larger peers. Examining the global distribution of impact evaluations for international development interventions, Cameron et al. (2016) note that the 10 most populated LMICs accounted for 41.3 % of all studies identified between 1981 and 2012. Although they note that S&IS are the most ‘densely’ studied countries – as measured by studies per 1 million population – there exists vast inequalities in evidence generation between large and small states. Another way, this lack of evidence materialises is seen in Gabani et al. (2022), who examines the influence of health financing systems on health system outcomes across countries. They remove “small and island countries, given that governance, health systems and health financing for those countries present peculiarities when compared to other countries.”^16^; Although removed for legitimate methodological considerations, this highlights how S&IS can even be overlooked in health system research. Overall, the health systems of such states remains poorly covered in the literature.

Finally, despite much evidence suggesting that African S&IS have well-functioning health systems, these countries remain vulnerable to external shocks that can impact health financing, the performance of the health system and health outcomes. Fig. 1 illustrated the economic volatility of African S&IS, while it is difficult to picture how these countries will be able to reduce their reliance on imports for a number of crucial health system inputs. Additionally, because of their small populations, the spread of disease can very quickly impact significant proportions of the population leading to country-wide impacts. McGuire et al. (2025) further explore whether African S&IS are building appropriate health system resilience against these vulnerabilities. However, even accounting for these structural vulnerabilities, it appears African S&IS are currently performing at least as well as their larger peers with respect to various measures of health system performance and health outcomes, and should focus on improving efficiency and resilience to sustain this.

## CRediT authorship contribution statement

**Ajoy Nundoochan:** Validation, Resources, Writing – review & editing. **Juliet Nabyonga-Orem:** Validation, Resources, Writing – review & editing. **Issiaka Sombie:** Resources, Writing – review & editing, Validation. **Paul Revill:** Funding acquisition, Resources, Writing – review & editing, Conceptualization, Project administration, Validation. **Simon Bland:** Funding acquisition, Validation, Resources, Writing – review & editing. **Edward Kataika:** Writing – review & editing, Resources, Validation. **Finn McGuire:** Formal analysis, Software, Writing – review & editing, Data curation, Methodology, Writing – original draft, Conceptualization. **Megha Rao:** Writing – review & editing, Validation, Resources. **Sakshi Mohan:** Resources, Writing – review & editing, Validation.

## Code availability

All code available upon request

## Funding

This study was supported by funding received from the Global Institute for Disease Elimination (GLIDE) as part of the Thanzi Labwino (Better Health) research project.

## Declaration of Competing Interest

The authors have no interests to declare

## Data Availability

All data available upon request

## Footnotes

1 For general information on the economic, social and environmental factors that contribute to structural vulnerabilities and development challenges facing the S&IS see Prasad (2009) and Bertram (2011).

2 Both qualitative and quantitative approaches have been proposed, see Maas (2009), Brito (2015), Kurecic et al. (2017) and Gussen (2019) for broader discussions of how S&IS may be identified. As absolute population is not an inherent feature of states and changes over time, that the countries classified as S&IS may also evolve over time. This is similar to how countries in the commonly used fragile or conflict-affected situations (FCS) categorisation can change.

3 However, the WB SSF includes many countries beyond the 1.5 million threshold, while the Commonwealth includes “countries with a bigger population but which share many of the same characteristics”. The IMF SDS status is population based but excludes advanced economies (i.e. Cyprus, Estonia, Bahrain) and fuel exporters (i.e. Brunei Darussalam and Equatorial Guinea) as classified by the World Economic Outlook.

4 Despite being presented as sharing commonalities in terms of their small land areas and population sizes. Haiti, Cuba and the Dominican Republic have populations > 10 million, while Namibia, Botswana, Papua New Guinea and Gabon are each geographically larger than the mean size of countries in Europe.

5 There are 54 independent states in Africa with a total continental population of 1424,810,790 (WDI, 2022).

6 For 2020 realised health expenditure data was not available for all countries. Therefore, 2020 is a combination of actual and modelled health expenditure data (WHO, 2022).

7 The dataset does not capture information on private for-profit facilities.

8 Social health insurance (SHI) is differentiated from national health insurance, which is universal regardless of contribution, while SHI may only be compulsory for certain groups to become members and only those making contributions are entitled to benefit from the scheme.

9 They examine 32 UN SIDS, excluding high-income states and dependent territories from the original list of 52.

10 https://www.afro.who.int/news/sids-pooled-procurement-initiative-improve-access-quality-medicines [accessed September 2023].

11 The AfCFTA Pharmaceutrical Initiative is a joint project between the United Nations Economic Commission for Africa (ECA), African Union Commission (AUC), African Union Development Agency (AUDA-NEPAD), Intergovernmental Authority on Development (IGAD), WHO and UNAIDS. The initiative was launched in 10 pilot countries; Seychelles, Madagascar, Comoros, Mauritius, Djibouti, Eritrea, Rwanda, Sudan, Kenya and Ethiopia.

12 Algeria (2019), Libya (2012), Morocco (2010), Lesotho (2012), Mauritius (1973), Seychelles (2012), Tunisia (2012) (WHO, Global Malaria Programme).

13 An alternative explanation is that differences in development levels among S&IS and non-S&IS are matched by differences in health aid which has been suggested is highly fungible (Dieleman et al. 2013).

14 Non-hospital/other health infrastructure may include health centres, medical centres, polyclinics, health posts, dispensaries, clinics, health huts, health units etc. Maina et al. (2019) not that there is no standardised definition of facility types making cross-country classifications and comparisons difficult for lower tier facilities.

15 Although it should be noted that, regardless of context, CHWs have key contributions towards strengthening primary health care.

16 Specifically, they note that there are frequent changes in the health financing system of S&IS compared to their larger peers.
