# Supplementary material for: Health financing and systems in African small and island states: Unique challenges and opportunities in achieving universal health coverage
Source: SSM Health Syst. 2025 Dec;5:100104. doi: 10.1016/j.ssmhs.2025.100104 (PMC12679919; doi:10.1016/j.ssmhs.2025.100104)
Supplement: Supplementary file 1 — Supplementary material [file mmc1.docx]

**Appendix**

**Appendix A: Multilateral Initiatives focused on Small & Island State issues**

**The Alliance of Small Island States (AOSIS)**

Founded in 1990, the intergovernmental organisation consists of 39 members and 5 observers. Primarily an advocacy body aimed at amplifying the voices of members to address shared concerns.

**United Nations Small Island Developing States (SIDS)**

Established in 1992, UNSID includes 39 states and 18 associate members of the UN (non-states) with an aggregate population of 65 million people. Despite the name, it includes four non-island countries facing similar economic and environmental challenges.

**World Bank Small States Forum (SSF)**

Established in 2000, the SSF consists of 50 member states, “42 countries classified as Small States according to the Bank Group definition (i.e., those with a population of 1.5 million or less) and eight other Small States Forum members with a population greater than 1.5 million that share similar challenges.” (**REF**) The SSF aims to raise the profile of the unique development challenges of members with annual meetings acting to bring their views to the attention of the international community.

**International Monetary Fund Small Developing States (SDS)**

Recognising the higher vulnerabilities facing small countries, in 2010 the IMF approved a formal categorisation of small states allowing exceptional eligibility and access to concessional financing. 34 member countries are defined as Small Developing States (SDS), representing 18% of the IMFs 190 member countries. Other than financing facilities, the IMF predominately provides policy guidance to support SDS in achieving sustainable economic growth.

**The Commonwealth Secretariat Small States**

Thirty three of the 56 Commonwealth members are small states. The organisation was an early pioneer of work and advocacy for small states dating back to 1977 (Sutton, 2001). The Secretariat organises the Commonwealth Ministerial Meeting on Small States and publishes the periodic *Small State Matters^^[[1]](#footnote-1)^^.*

| **Small and Island State International Initiative Membership** | | | | | |
| --- | --- | --- | --- | --- | --- |
| **Countries** | **World Bank SSF** | **UN SIDS** | **Commonwealth Small States Secretariat** | **IMF SDS** | **The Alliance of Small Island States (AOSIS)** |
| **Africa** |  |  |  |  |  |
| Botswana | x |  | x |  |  |
| Cabo Verde | x | x |  | x | x |
| Comoros | x | x |  | x | x |
| Djibouti | x |  |  | x |  |
| Equatorial Guinea | x |  |  |  |  |
| Eswatini | x |  | x | x |  |
| Gabon | x |  | x |  |  |
| The Gambia | x |  | x |  |  |
| Guinea-Bissau | x | x |  |  | x |
| Lesotho | x |  | x |  |  |
| Mauritius | x | x | x | x | x |
| Namibia | x |  | x |  |  |
| Sao Tome & Principe | x | x |  | x | x |
| Seychelles | x | x | x | x | x |
| **Asia** |  |  |  |  |  |
| Bhutan | x |  |  | x |  |
| Brunei Darussalam | x |  | x |  |  |
| Maldives | x | x | x | x | x |
| Singapore |  | x | x |  | x |
| Timor-Leste | x | x |  | x | x |
| **Europe** |  |  |  |  |  |
| Cyprus | x |  | x |  |  |
| Estonia | x |  |  |  |  |
| Iceland | x |  |  |  |  |
| Malta | x |  | x |  |  |
| Montenegro | x |  |  |  |  |
| San Marino | x |  |  |  |  |
| **Caribbean** |  |  |  |  |  |
| Antigua & Barbuda | x | x | x | x | x |
| The Bahamas | x | x | x | x | x |
| Barbados | x | x | x | x | x |
| Cuba |  | x |  |  | x |
| Dominica | x | x | x | x | x |
| Dominican Republic |  | x |  |  | x |
| Grenada | x | x |  | x | x |
| Haiti |  | x |  |  | x |
| Jamaica | x | x | x |  | x |
| St. Kitts & Nevis | x | x | x | x | x |
| St. Lucia | x | x | x | x | x |
| St. Vincent & The Grenadines | x | x | x | x | x |
| Trinidad & Tobago | x | x | x | x | x |
| Anguilla |  | x |  |  |  |
| Aruba |  | x |  |  |  |
| Bermuda |  | x |  |  |  |
| British Virgin Islands |  | x |  |  |  |
| Cayman Islands |  | x |  |  |  |
| Curacao |  | x |  |  |  |
| Guadeloupe |  | x |  |  |  |
| Martinique |  | x |  |  |  |
| Montserrat |  | x |  |  |  |
| Puerto Rico |  | x |  |  | Observer |
| Sint Maarten |  | x |  |  | Observer |
| Turks and Caicos Islands |  | x |  |  |  |
| U.S. Virgin Islands |  | x |  |  | Observer |
| **Central and South America** |  |  |  |  |  |
| Belize | x | x | x | x | x |
| Guyana |  |  |  |  |  |
| Suriname |  |  |  |  |  |
| **Middle East** |  |  |  |  |  |
| Bahrain | x | x |  |  |  |
| Qatar | x |  |  |  |  |
| **Pacific Region** |  |  |  |  |  |
| Fiji | x | x | x | x | x |
| Kiribati | x | x | x | x | x |
| Marshall Islands | x | x |  | x | x |
| Micronesia | x | x |  | x | x |
| Nauru | x | x | x | x | x |
| Palau | x | x |  | x | x |
| Papua New Guinea |  | x | x |  | x |
| Samoa | x | x | x | x | x |
| Solomon Islands | x | x | x | x | x |
| Tonga | x | x | x | x | x |
| Tuvalu | x | x | x | x | x |
| Vanuatu | x | x | x | x | x |
| American Samoa |  | x |  |  | Observer |
| Commonwealth of Northern Marianas |  | x |  |  |  |
| Cook Islands |  | x |  |  |  |
| French Polynesia |  | x |  |  |  |
| Guam |  | x |  |  | Observer |
| New Caledonia |  | x |  |  |  |
| Niue |  | x |  |  |  |

**Appendix B: Incidence of household catastrophic health expenditure**

| **Appendix Table B: Financial Risk Protection measured by CHE at 10% and 25%** | | |
| --- | --- | --- |
| Country | Incidence of catastrophic health spending (10% of household spending threshold) | Incidence of catastrophic health spending (25% of household spending threshold) |
| Botswana | 4.32 | 1.01 |
| Cabo Verde | 2.05 | 0.02 |
| Comoros | 8.81 | 1.64 |
| Djibouti | - | - |
| Eswatini | 5.00 | 1.29 |
| Equatorial Guinea | - | - |
| Gabon | 3.83 | 0.72 |
| Gambia | 0.20 | 0.03 |
| Guinea-Bissau | 5.05 | 0.37 |
| Lesotho | 4.51 | 1.35 |
| Mauritius | 8.20 | 1.9 |
| Namibia | 1.52 | 0.32 |
| São Tomé and Principe | 4.83 | 1.15 |
| Seychelles | 2.56 | 1.3 |
| AU S&IS Mean | 4.24 | 0.93 |
| AU Non-S&IS Mean | 7.91 | 1.86 |
| AU Mean | 6.91 | 1.61 |
| AU S&IS Countries | 12 | 12 |
| AU Non-S&IS Countries | 32 | 32 |
| AU S&IS Average Year Data | 2014.5 | 2014.5 |
| AU non-S&IS Average Year Data | 2016.4 | 2016.4 |
| AU Average Year Data | 2015.9 | 2015.9 |
| **Notes:** Data from the WHO Global Health Observatory Database | | |

**Appendix C:**

**Appendix Table C** illustrates countries progress towards achieving UHC, as measured by the GBD UHC Effective Coverage Index.

| **Appendix Table C: Universal Health Care Effective Coverage** | | | |
| --- | --- | --- | --- |
| **Country** | **UHC Effective Coverage 1990** | **UHC Effective Coverage 2010** | **UHC Effective Coverage 2019** |
| Botswana | 44.6 [40.2 - 48.8] | 46.5 [42.1 - 51.5] | 57.5 [52.5 - 62.4] |
| Cape Verde | 49.8 [47.4 - 52.3] | 61.2 [59.2 - 63.1] | 62.2 [58.6 - 65.1] |
| Comoros | 35.8 [27.3 - 54] | 41.7 [36.8 - 46.6] | 48.1 [42.7 - 53.8] |
| Djibouti | 38.9 [32.8 - 44.6] | 40.5 [34.5 - 47.1] | 45.3 [38.7 - 52] |
| Eswatini | 44.8 [40.7 - 49] | 35 [30.8 - 39.5] | 53.4 [48.4 - 58.6] |
| Equatorial Guinea | 16.8 [12.1 - 21.9] | 42.5 [35.7 - 49.4] | 50 [42.3 - 56.3] |
| Gabon | 35.8 [31.6 - 40] | 41.9 [38.4 - 45.9] | 53 [48.4 - 58.1] |
| The Gambia | 47.6 [41.1 - 54.2] | 45.9 [42.3 - 49.9] | 48.1 [43.5 - 53.5] |
| Guinea-Bissau | 18.8 [14.6 - 23.3] | 23.9 [20.3 - 28.2] | 35.7 [30.4 - 41.5] |
| Lesotho | 39.4 [35.4 - 43.4] | 31.9 [28.3 - 35.6] | 38.7 [33.9 - 44.3] |
| Mauritius | 43.8 [41.5 - 46] | 49 [47 - 51] | 55.8 [51.3 - 60] |
| Namibia | 43.9 [39.9 - 48.8] | 49.2 [45.6 - 53] | 62.2 [57.4 - 66.8] |
| São Tomé and Principe | 41.9 [38 - 48] | 51.1 [48.3 - 54.4] | 54.8 [50.7 - 59.1] |
| Seychelles | 53.9 [52 - 55.8] | 60.2 [58.5 - 61.8] | 61.5 [59.5 - 63.5] |
| **AU S&IS Mean** | 28.7 [24.4 - 33.5] | 44.3 [40.5 - 48.4] | 51.9 [47 - 56.8] |
| **AU non-S&IS Mean** | 32.8 [28.5 - 37.6] | 38.6 [34.5 - 42.9] | 46.8 [41.7 - 52.2] |
| **AU Mean** | 31.7 [27.4 - 36.5] | 40.1 [36.1 - 44.3] | 48.2 [43.1 - 53.4] |
| **Notes:**Brackets represent 95% uncertainty interval. | | | |

It’s notable that AU S&IS have lower UHC effective coverage in 1990 compared to non-S&IS, but have since, on average, overtaken their larger neighbours. However, there remains significant heterogeneity within the S&IS group as Djibouti, Guinea-Bissau and Lesotho have UHC effective coverage indices below the non-S&IS average, while Cape Verde, Namibia and Seychelles far exceed this level.

**Appendix** **E:**

**Appendix Figure E: Change in THE per capita and UHC Effective Coverage Index**


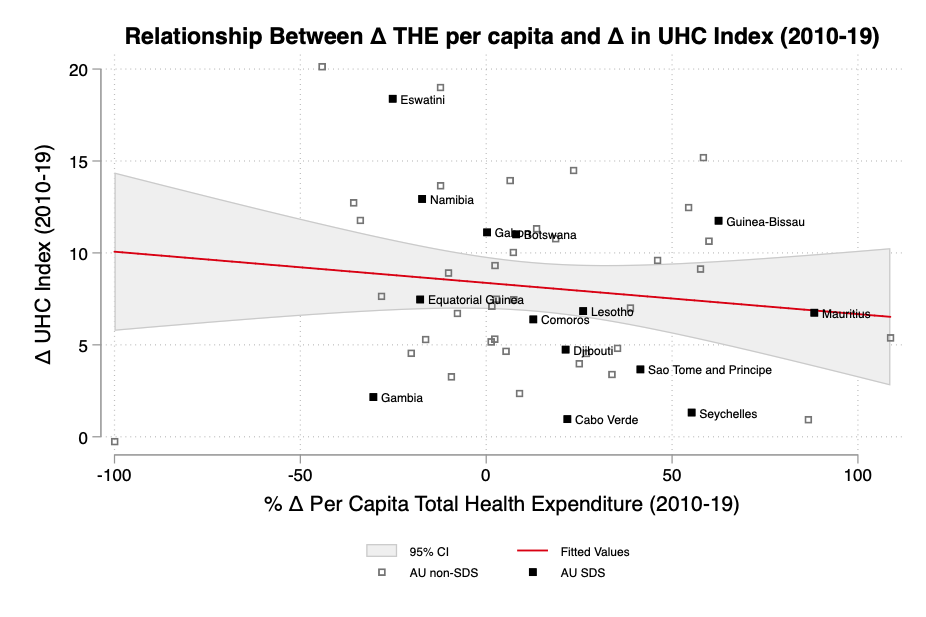


**Appendix F: HAQ Index variation by age groups**

| **Appendix Table D: Global Burden of Disease Health Care Access & Quality (HAQ) Index** | | | | | | | | | | | | |
| --- | --- | --- | --- | --- | --- | --- | --- | --- | --- | --- | --- | --- |
| **Country** | **Overall Health Care Access & Quality Index 1990** | **Overall Health Care Access & Quality Index 2019** | **Change** | **Young Health Care Access & Quality Index 1990** | **Young Health Care Access & Quality Index 2019** | **Change** | **Working Health Care Access & Quality Index 1990** | **Working Health Care Access & Quality Index 2019** | **Change** | **Post-working Health Care Access & Quality Index 1990** | **Post-working Health Care Access & Quality Index 2019** | **Change** |
| Botswana | 27.4 [24 - 31.2] | 37.5 [33.4 - 42] | 10.1 | 46.4 [42.6 - 50.3] | 51.6 [46.7 - 56.5] | 5.2 | 26.6 [22 - 31.9] | 37 [31.6 - 43.2] | 10.4 | 23 [18.8 - 27.7] | 30.2 [26.2 - 34.8] | 7.2 |
| Cape Verde | 36.2 [34.3 - 38.4] | 50.2 [47.7 - 52.9] | 14.1 | 43.4 [39.7 - 47.5] | 65.7 [61.7 - 70.2] | 22.3 | 40.2 [37.7 - 42.4] | 51.9 [49 - 54.6] | 11.6 | 39.4 [36.4 - 42.4] | 45.4 [42.3 - 48.6] | 6.0 |
| Comoros | 21.5 [16 - 35.3] | 31.8 [28.8 - 36.2] | 10.3 | 30.6 [23.9 - 46.1] | 45 [39.2 - 50.9] | 14.4 | 27.3 [19.9 - 44.5] | 35.6 [30.9 - 41.8] | 8.3 | 22.2 [16.8 - 31.8] | 28.7 [24.9 - 32.4] | 6.5 |
| Djibouti | 23.6 [20.2 - 27.5] | 32.6 [27.5 - 37.8] | 9.0 | 30 [25.2 - 35.4] | 40.9 [34.7 - 47.2] | 10.9 | 29.3 [24.4 - 34.6] | 36.3 [29.7 - 43.3] | 7.0 | 25.3 [21 - 29.6] | 30.6 [25.6 - 35.5] | 5.4 |
| Eswatini | 27.4 [24.3 - 30.9] | 32.5 [28.3 - 36.8] | 5.1 | 39.8 [35.1 - 44.5] | 47.7 [43.8 - 51.5] | 7.9 | 29.1 [24.8 - 33.7] | 32 [26.2 - 38.2] | 2.9 | 26.5 [21.5 - 31.3] | 28.6 [24.2 - 32.6] | 2.2 |
| Equatorial Guinea | 12.3 [8.7 - 16.1] | 42.4 [35.4 - 48.8] | 30.1 | 21 [15.9 - 27.5] | 59.1 [50.2 - 67.7] | 38.1 | 14.5 [9.7 - 19.4] | 44.9 [37.1 - 51.6] | 30.4 | 16.9 [11.9 - 22.4] | 38.1 [31.9 - 44.4] | 21.2 |
| Gabon | 24.5 [21.1 - 28] | 39.6 [35.7 - 44.1] | 15.1 | 34.9 [29 - 41.6] | 57.2 [49.5 - 64.4] | 22.4 | 27.9 [23.3 - 32.1] | 41.5 [36.3 - 46.8] | 13.5 | 24.6 [20.3 - 29.2] | 35.3 [30.8 - 40.6] | 10.7 |
| The Gambia | 27.3 [22.9 - 31.9] | 34.7 [31.4 - 39] | 7.4 | 30.3 [24.2 - 36.8] | 47.6 [41.5 - 53.7] | 17.3 | 32.5 [27 - 38.6] | 36.8 [32.3 - 41.9] | 4.3 | 28.8 [23.6 - 34.3] | 31.7 [27.3 - 37.2] | 2.9 |
| Guinea-Bissau | 13.6 [9.6 - 18.3] | 24.3 [20.6 - 27.8] | 10.6 | 17.5 [11.6 - 25.1] | 36.6 [31.4 - 41.7] | 19.2 | 17.4 [13 - 22.5] | 25.7 [21.7 - 29.9] | 8.3 | 16.6 [11.8 - 22] | 23.2 [19.2 - 27.2] | 6.6 |
| Lesotho | 25.4 [22.2 - 29.2] | 26.3 [22.2 - 30.5] | 0.9 | 39.9 [36.4 - 43.8] | 44.7 [40.5 - 48.4] | 4.8 | 27.4 [23.5 - 31.9] | 25.2 [20.1 - 30.8] | -2.2 | 24.5 [19.4 - 30.1] | 22.5 [17.4 - 27] | -2.0 |
| Mauritius | 45.4 [43.8 - 46.9] | 56.7 [53.5 - 59.6] | 11.2 | 62.2 [60.4 - 63.8] | 70.5 [67.8 - 73] | 8.3 | 43.1 [41.3 - 44.8] | 53.8 [50.3 - 57] | 10.7 | 41.2 [39.1 - 43.3] | 56.9 [53.6 - 60.1] | 15.7 |
| Namibia | 25.7 [22.5 - 30] | 39.9 [35.5 - 44.9] | 14.3 | 41.9 [36.3 - 50.2] | 57.5 [52.2 - 63.8] | 15.6 | 25.7 [21.6 - 31.5] | 39.1 [33.8 - 44.8] | 13.4 | 23.5 [19.6 - 27.5] | 32 [27.9 - 36.6] | 8.5 |
| São Tomé and Principe | 28 [25.3 - 31.9] | 41.4 [37.5 - 45.2] | 13.4 | 26 [22.1 - 30] | 54 [48.3 - 58.6] | 28.0 | 34.7 [30.4 - 40.5] | 43.1 [38.2 - 47.3] | 8.4 | 30.5 [26.7 - 34.5] | 35.6 [31.5 - 39.4] | 5.1 |
| Seychelles | 39.4 [37.8 - 41.1] | 52.8 [50.6 - 55] | 13.4 | 62.9 [60.6 - 65.5] | 70.3 [67.4 - 73.2] | 7.4 | 38.2 [36 - 40.3] | 52.2 [50 - 54.4] | 14.0 | 34.6 [32.1 - 37.2] | 47.7 [45 - 50.5] | 13.0 |
| **AU S&IS Mean** | 27 [23.8 - 31.2] | 38.8 [34.9 - 42.9] | 11.8 | 37.6 [33.1 - 43.4] | 53.5 [48.2 - 58.6] | 15.8 | 29.6 [25.3 - 34.9] | 39.7 [34.8 - 44.7] | 10.1 | 27 [22.8 - 31.7] | 34.8 [30.6 - 39.1] | 7.8 |
| **AU non-S&IS Mean** | 22.3 [19.2 - 25.9] | 34 [30 - 38.2] | 11.6 | 25.9 [21 - 32] | 42.9 [37.2 - 48.8] | 16.9 | 27.9 [23.8 - 32.4] | 37.2 [32.3 - 42.4] | 9.2 | 26.3 [22.1 - 30.9] | 33.1 [28.6 - 37.8] | 6.8 |
| **AU Mean** | 23.5 [20.4 - 27.3] | 35.2 [31.3 - 39.4] | 11.7 | 29 [24.1 - 34.9] | 45.6 [40 - 51.3] | 16.6 | 28.4 [24.2 - 33] | 37.8 [32.9 - 43] | 9.4 | 26.4 [22.2 - 31.1] | 33.5 [29.1 - 38.2] | 7.1 |
|  | | | | | | | | | | | | |

1. in 2000 a joint World Bank and Commonwealth Secretariat commissioned Task Force released a document – *Small States: Meeting Challenges in the Global Economy* – outlining a way-forward for S&IS. This report was followed by *Towards an Outward-Oriented Development Strategy for Small States* (Briguglio et al. 2005). [↑](#footnote-ref-1)
